# AGR2 and FOXA1 as prognostic markers in ER-positive breast cancer

**DOI:** 10.1186/s12885-023-10964-6

**Published:** 2023-08-11

**Authors:** Meng Zhou, Xing-li Gan, Yue-xiang Ren, Qian-xin Chen, Yuan-zhong Yang, Zi-jin Weng, Xiao-fang Zhang, Jie-xia Guan, Lu-ying Tang, Ze-fang Ren

**Affiliations:** 1 School of Public Health, Sun Yat-Sen University, 74 Zhongshan 2Nd Rd, Guangzhou, 510080 China; 2 The Third Affiliated Hospital, Sun Yat-Sen University, 600 Tianhe Rd, Guangzhou, 510630 China; 3 The Sun Yat-sen University Cancer Center, Guangzhou, China

**Keywords:** Breast cancer, Prognosis, AGR2, FOXA1

## Abstract

**Background:**

The prognostic role of either forkhead box A1 (FOXA1) or anterior gradient 2 (AGR2) in breast cancer has been found separately. Considering that there were interplays between them depending on ER status, we aimed to assess the statistical interaction between AGR2 and FOXA1 on breast cancer prognosis and examine the prognostic role of the combination of them by ER status.

**Methods:**

AGR2 and FOXA1 expression in tumor tissues were evaluated with tissue microarrays by immunohistochemistry in 915 breast cancer patients with follow up data. The expression levels of these two markers were treated as binary variables, and many different cutoff values were tried for each marker. Survival and Cox proportional hazard analyses were used to evaluate the relationship between AGR2, FOXA1 and prognosis, and the statistical interaction between them on the prognosis was assessed on multiplicative scale.

**Results:**

Statistical interaction between AGR2 and FOXA1 on the PFS was significant with all the cutoff points in ER-positive breast cancer patients but not ER-negative ones. Among ER-positive patients, the poor prognostic role of the high level of FOXA1 was significant only in patients with the low level of AGR2, and vice versa. When AGR2 and FOXA1 were considered together, patients with low levels of both markers had significantly longer PFS compared with all other groups.

**Conclusions:**

There was a statistical interaction between AGR2 and FOXA1 on the prognosis of ER-positive breast cancer. The combination of AGR2 and FOXA1 was a more useful marker for the prognosis of ER-positive breast cancer patients.

**Supplementary Information:**

The online version contains supplementary material available at 10.1186/s12885-023-10964-6.

## Background

Forkhead box A1 (FOXA1), a member of FOX family of transcription factors, can bind to the promoters of many genes associated with the carcinogenesis and cancer development [1–3]. In breast cancer, it can act as both a growth stimulator and a repressor. For example, it enhances binding of estrogen receptor (ER) to its target genes and promotes the progression of the tumor [4], while it increases the expression levels of tumor suppressor genes such as E-cadherin and p27, which prevents the progression of breast cancer [5–7].

For the prognostic roles of FOXA1 in breast cancer, the results were quite different with positive (high expression of FOXA1 related to a better prognosis) [8, 9], or negative associations [10–12]. Notably, previous studies found that the prognostic roles of FOXA1 could be affected by other factors, such as androgen receptor (AR) status, and the combined marker of FOXA1 and AR was found as superior predicting marker of the prognosis compared to the single FOXA1 or AR in breast cancer [9, 13, 14]. Intriguingly, the recent study reported that FOXA1 exerted its biological function by regulating anterior gradient 2 (AGR2, a protein belonging to the family of Protein Disulfide Isomerase) expression [15], and both AGR2 and FOXA1 were needed in the ER signaling [16], suggesting the biological role of FOXA1 may be affected by AGR2. For the prognostic roles of AGR2 in breast cancer, the findings were also inconsistent with positive and negative associations [17–20], suggesting the role of AGR2 may also be affected by other factors. Therefore, it remains to be explored whether there is a statistical interaction between AGR2 and FOXA1 on the prognosis of breast cancer.

In the present study, therefore, the statistical interaction between FOXA1 and AGR2 on breast cancer prognosis was explored. Moreover, we aimed to examine the association of the combination of AGR2 and FOXA1 with the prognosis of breast cancer.

## Materials and methods

### Study population

**Fig. 1:**
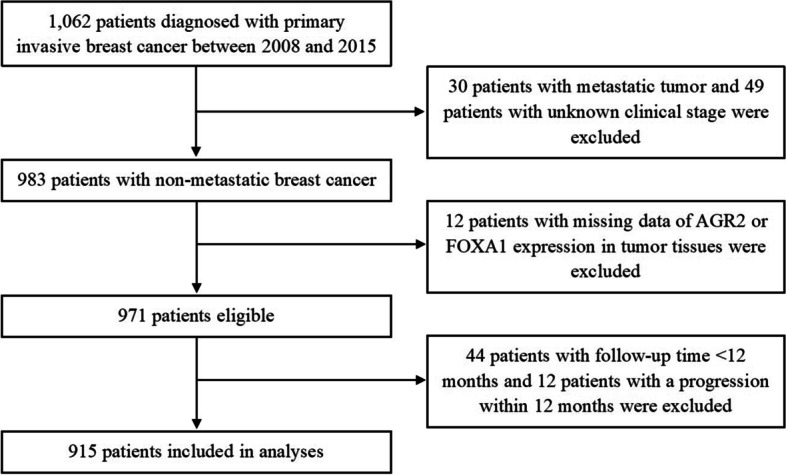
Flow chart of the study cohort

A total of 1062 women with pathologically diagnosed primary invasive breast cancer (Both ER-positive and ER-negative) between January 2008 and December 2015 and > 1 cm of tumor size in diameter were recruited from the Cancer Center of Sun Yat-sen University in Guangzhou, China. Patients with metastatic tumor and missing information of clinical stage and the expression levels of AGR2 and FOXA1 in tumor tissues were excluded, and 971 patients were eligible (Fig. 1). This study was approved by the Ethics Committee of the School of Public Health at Sun Yat-sen University. Informed consent was obtained from each participant.

### Baseline data collection

Information on clinicopathological characteristics was collected at diagnosis from patients’ medical records, including age, histological grade, clinical stage, estrogen receptor (ER), progesterone receptor (PR) and human epidermal growth factor receptor 2 (HER2) status etc. The status of ER, PR, and HER2 was defined according to American Society of Clinical Oncology/College of American Pathologists (ASCO/CAP) guidelines [21, 22].

### Tissue microarray construction and immunohistochemical analysis

The expression levels of AGR2 and FOXA1 were evaluated with tissue microarrays (TMAs) by immunohistochemistry (IHC). TMAs were constructed as previously described [23]. Briefly, formalin-fixed and paraffin-embedded tissues of included patients were retrieved. Hematoxylin and eosin (HE)-stained sections of tissue specimens were reviewed by two experienced pathologists, followed by re-slicing and re-staining with HE. Representative tumor tissue regions and adjacent normal tissue regions (If available) were marked on the re-stained HE sections. From the marked regions, two tumor tissue cylinders and one adjacent normal tissue cylinder (If unavailable, it would be replaced with the tumor tissue) with a diameter of 1 mm were punched out of the corresponding paraffin block as donor block and placed into the TMA paraffin block using an automatic tissue arrayer.

The TMAs were heated at 60 °C for 2 h and then cleared with xylene and ethanol. Then antigen retrieval was accomplished using EDTA (PH 9.0) in super-pressure kettle and endogenous peroxide was blocked using 3% H2O2. Antigen–antibody reactions for AGR2 and FOXA1 were performed separately. For AGR2, slides were incubated in rabbit monoclonal to AGR2 [EPR3278] (ab76473, diluted 1:200, Abcam) and then labeled with the EnVision Detection System (Peroxidase/DAB, Rabbit/Mouse) (Dako K5007). For FOXA1, slides were incubated in rabbit monoclonal to FOXA1 [EPR10881]-ChIP Grade (ab170933, diluted 1:100, Abcam). Then slides were developed by diaminobenzidine (DAB) and counterstained by hematoxylin. These slides were finally dehydrated and mounted. We verified the antibodies with the positive samples which mentioned in the protocol before the formal testing (Human colon tissue for AGR2; Human breast carcinoma tissue for FOXA1). The results showed that all of these positive controls expressed the corresponding proteins.

**Fig. 2:**
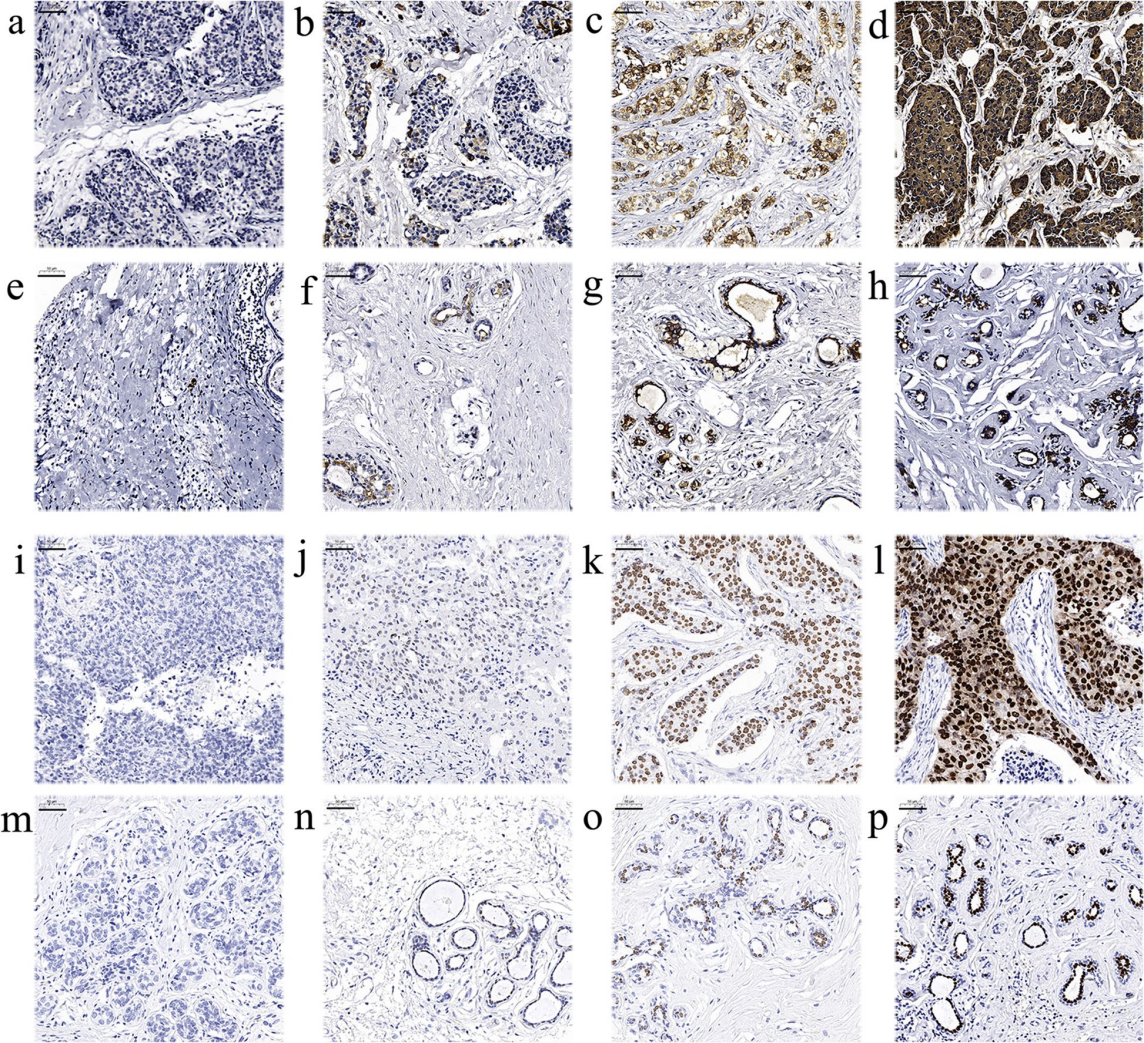
Representative immunohistochemical staining of AGR2 and FOXA1 in both tumor and adjacent tissues. **a**–**d**: The intensity of AGR2 in tumor tissues (**a**, no staining; **b**, weak; **c**, moderate; **d**, strong). **e**–**h**: The intensity of AGR2 in adjacent tissues (**e**, no staining; **f**, weak; **g**, moderate; **h**, strong). **i**–**l**: The intensity of FOXA1 in tumor tissues (**i**, no staining; **j**, weak; **k**, moderate; **l**, strong). **m**–**p**: The intensity of FOXA1 in adjacent tissues (**m**, no staining; **n**, weak; **o**, moderate; **p**, strong). Scale bar indicates 50 μm

IHC stained sections were digitally imaged using Pannoramic Scanner and CaseViewer software. IHC staining for each of these markers was evaluated by an experienced pathologist and scored for staining intensity (0-no staining, 1-weak, 2-moderate and 3-strong) and percentage of tumor cell (cytoplasmic staining for AGR2; nuclear staining for FOXA1) staining (0–100). Staining intensity and the percentage of positive cells were multiplied to generate IHC scoring (H-score) ranging from 0 to 300. Mean value of duplicate scores was adapted for analysis. Representative IHC staining of AGR2 and FOXA1 in both tumor and adjacent tissues was shown in Fig. 2.

### Follow up and outcome

Patients were followed up by phone calls or out-patient visits every 3 months in the first year, every 6 months in the second and third year after diagnosis and annually thereafter. Outcome of interest was progression-free survival (PFS). PFS was defined as the time from diagnosis to disease progression including recurrence, metastasis, and death. Survival status was censored at the latest follow-up date or Dec 31, 2021. Of 971 eligible women, 44 patients with follow-up time < 12 months and 12 patients with a progression within 12 months were excluded, and 915 patients were included in the statistical analysis (Fig. 1).

### Statistical analysis

Wilcoxon signed rank test was used to compare the expression levels of AGR2 and FOXA1 between tumor tissues and adjacent tissues. Spearman’s correlation test was used to compare the correlation coefficient (*r*) of AGR2 and FOXA1 in both tumor and adjacent tissues. Kruskal–Wallis test and Mann–Whitney U test were used to test the associations of AGR2 and FOXA1 H-scores (defined as a continuous variable) with age, histological grade, tumor size, nodal status, clinical stage and the status of ER, PR and HER2. Then the H-score of AGR2 and FOXA1 was treated as binary variable. Kaplan–Meier method was used to estimate the 5-year PFS rate. Cox proportional hazard model was used to estimate Hazard ratios (HRs) and their 95% confidence intervals (CIs) for the associations between various prognostic variables and PFS. Multiplicative scale was used to estimate the statistical interaction between AGR2 and FOXA1 on the PFS.

Many methods have been performed to define the cutoff point for the low /negative and high/positive expression for AGR2 and FOXA1 previously [9, 10, 24–26]. For both AGR2 and FOXA1, in this study, we first used quantiles to divide the patients into two subgroups with the low and high expression level: median (low vs. high), tertiles (lowest tertiles vs. the rest) and quartiles (lowest quartiles vs. the rest) were used, respectively. In addition, the optimal cutoff point was determined by the minimum *P* value from log-rank chi-square statistics based on PFS using the X-tile 3.6.1 software (Yale University, New Haven, CT, USA) for both AGR2 and FOXA1 [27]. All analyses were done using SPSS version 21 and R version 4.1.2 and a two-sided *P*-value below 0.05 was considered as statistical significance.

## Results

### High expression level of AGR2 and FOXA1 in tumor tissues

**Fig. 3:**
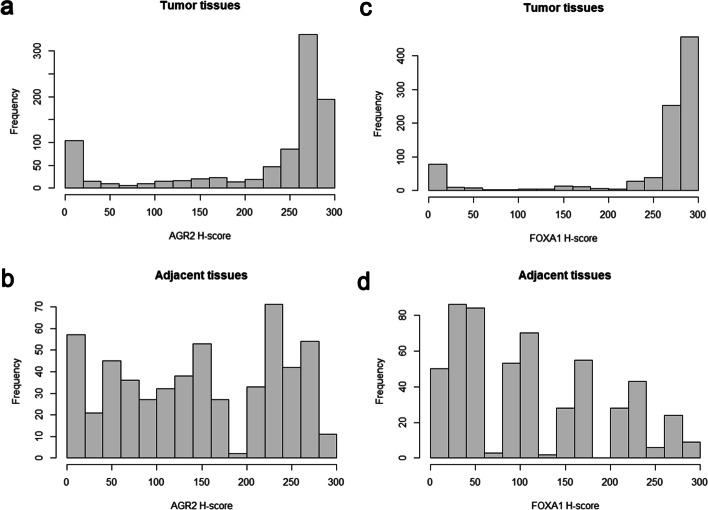
Histogram of the distribution of AGR2 and FOXA1 H-score in tumor and adjacent tissues of breast cancer. **a**, **b**: AGR2 H-score in tumor (**a**) and adjacent (**b**) tissues. **c**, **d**: FOXA1 H-score in tumor (**c**) and adjacent (**d**) tissues

Of 915 women included in the analysis, distributions of AGR2 and FOXA1 H-scores in tumor and adjacent tissues were shown in Fig. 3. The data of AGR2 and FOXA1 in adjacent tissues was available for 549 and 541 patients, respectively. Therefore, the comparisons of AGR2 and FOXA1 levels between tumor tissues and adjacent tissues were performed among 549 and 541 patients, respectively. Median (P_25_, P_75_) of AGR2 H-scores in tumor tissues [270.0 (180.0, 270.0)] was higher than that in adjacent tissues [150.0 (80.0, 240.0)] (*P* < 0.001). The median (P_25_, P_75_) of FOXA1 H-scores in tumor tissues [277.5 (255.0, 285.0)] was also significantly higher than that in adjacent tissues [90.0 (40.0, 180.0)] (*P* < 0.001). There was also significant positive correlation between the expression levels of AGR2 and FOXA1 in both tumor tissues (*r* = 0.441, *P* < 0.001) and adjacent tissues (*r* = 0.567, *P* < 0.001).

### Clinicopathological characteristics and the associations with AGR2 and FOXA1

The median age at diagnosis was 48 years (interquartile range: 42–56 years) among 915 eligible patients. The majority of the women were diagnosed with low histological grade (grade I/II: 73.8%), early clinical stage (stage I/II: 72.3%), ER-positive (74.0%), PR-positive (73.0%), or HER2-negative (66.5%) (Table 1). Univariate analysis showed that tumor size, nodal status and clinical stage were associated with breast cancer PFS (Supplemental Table 1, see Additional file 1).

**Table 1:** Clinicopathological characteristics and the associations with AGR2 and FOXA1 in tumor tissues (*N* = 915)

Factors	*N* (%)	AGR2		FOXA1	
		H-score [Median (P25, P75)]	*P* value^a^	H-score [Median (P25, P75)]	*P* value^a^
Age (years)			0.632		0.359
≤ 40	200 (21.9)	270.0 (180.0, 270.0)		277.5 (260.0, 285.0)	
41–60	609 (66.6)	270.0 (200.0, 270.0)		280.0 (270.0, 285.0)	
> 60	106 (11.6)	270.0 (212.5, 270.0)		285.0 (270.0, 285.0)	
Histological grade			** < 0.001**		** < 0.001**
I/II	620 (73.8)	270.0 (230.8, 270.0)		285.0 (270.0, 285.0)	
III	220 (26.2)	255.0 (10.0, 270.0)		270.0 (13.1, 285.0)	
Missing	75				
Tumor size (cm)			0.964		0.876
≤ 2	282 (30.8)	270.0 (213.1, 270.0)		285.0 (270.0, 285.0)	
> 2	633 (69.2)	270.0 (190.0, 270.0)		280.0 (265.0, 285.0)	
Nodal status			0.177		0.245
Negative	421 (46.0)	270.0 (170.0, 270.0)		280.0 (262.5, 285.0)	
Positive	494 (54.0)	270.0 (218.1, 270.0)		285.0 (270.0, 285.0)	
Clinical stage			0.052		0.398
I	169 (18.5)	270.0 (165.0, 270.0)		277.5 (262.5, 285.0)	
II	493 (53.9)	270.0 (212.5, 277.5)		285.0 (270.0, 285.0)	
III	253 (27.7)	270.0 (180.0, 270.0)		280.0 (265.0, 285.0)	
ER			** < 0.001**		** < 0.001**
Negative	228 (26.0)	205.4 (0.0, 270.0)		270.0 (0.0, 285.0)	
Positive	650 (74.0)	270.0 (240.0, 277.5)		285.0 (270.0, 285.0)	
Missing	37				
PR			** < 0.001**		** < 0.001**
Negative	237 (27.0)	255.0 (35.0, 270.0)		277.5 (127.5, 285.0)	
Positive	641 (73.0)	270.0 (240.0, 270.0)		285.0 (270.0, 285.0)	
Missing	37				
HER2			**0.005**		0.123
Negative	614 (66.5)	270.0 (170.0, 270.0)		280.0 (260.0, 285.0)	
Equivocal	77 (8.2)	270.0 (220.0, 270.0)		285.0 (270.0, 285.0)	
Positive	224 (25.3)	270.0 (247.5, 271.9)		285.0 (270.0, 285.0)	

^a^ *P* value for Kruskal–Wallis test or Mann–Whitney U test

AGR2 expression was higher in tumors with histological grade I/II, ER-positive, PR-positive or HER2-positive than the expression in tumors with histological grade III, ER-negative, PR-negative or HER2-negative (all *P* < 0.05) (Table 1). Similar pattern of associations was found for FOXA1; the expression level of FOXA1 was higher in tumors with histological grade I/II, ER-positive or PR-positive than their counterparts (all *P* < 0.05) (Table 1).

### Independent prognostic effects of AGR2 and FOXA1

Of the 915 eligible patients, 194 experienced disease progression and death with a median follow-up time of 82.2 months (interquartile range: 56.8–119.7) and the five-year PFS rate was 83.8%. For ER-positive breast cancer, the cutoff points of AGR2 defined by median (cutoff 1), lowest tertiles (cutoff 2), lowest quartiles (cutoff 3) and the optimal cutoff point (cutoff 4) were 270.0, 260.0, 245.0 and 215.0, respectively; the cutoff points of FOXA1 were 285.0, 280.0, 275.0 and 255.0, respectively (Table 2). In multivariate analysis, there were significant associations between a poor PFS and the high expression level of both AGR2 [HR (95% CI) = 2.19 (1.25, 3.83)] and FOXA1 [HR (95% CI) = 1.99 (1.01, 3.93)] with the optimal cutoff point (Table 2).

**Table 2:** Hazard ratios for the associations between AGR2 and FOXA1 and ER-positive breast cancer PFS with various cutoff values (*N* = 650)

Markers	H-score	Events /Total	Crude HR (95%CI)	Adjusted HR (95%CI)^a^
AGR2				
Cutoff 1				
Median _low_	0–270.0	43 /238	1.00 (reference)	1.00 (reference)
Median _high_	270.0–300.0	89 /412	1.21 (0.84, 1.74)	1.13 (0.77, 1.65)
Cutoff 2				
Tertile1	0–260.0	36 /221	1.00 (reference)	1.00 (reference)
Tertile2-3	260.0–300.0	96 /429	1.43 (0.97, 2.10)	1.31 (0.88, 1.96)
Cutoff 3				
Quartile1	0–245.0	24 /165	1.00 (reference)	1.00 (reference)
Quartile2-4	245.0–300.0	108 /485	**1.64 (1.06, 2.56)**	1.48 (0.95, 2.33)
Cutoff 4				
Low	0–215.0	14 /125	1.00 (reference)	1.00 (reference)
High	215.0–300.0	118 /525	**2.26 (1.30, 3.94)**	**2.19 (1.25, 3.83)**
FOXA1				
Cutoff 1				
Median _low_	0–285.0	52 /280	1.00 (reference)	1.00 (reference)
Median _high_	285.0–300.0	80 /370	1.16 (0.82, 1.65)	1.14 (0.79, 1.65)
Cutoff 2				
Tertile1	0–280.0	51 /261	1.00 (reference)	1.00 (reference)
Tertile2-3	280.0–300.0	81 /389	1.08 (0.76, 1.54)	1.05 (0.73, 1.52)
Cutoff 3				
Quartile1	0–275.0	32 /177	1.00 (reference)	1.00 (reference)
Quartile2-4	275.0–300.0	100 /473	1.20 (0.80, 1.78)	1.23 (0.81, 1.86)
Cutoff 4				
Low	0–255.0	10 /72	1.00 (reference)	1.00 (reference)
High	255.0–300.0	122 /578	1.76 (0.92, 3.35)	**1.99 (1.01, 3.93)**

Cutoff 1, median; Cutoff 2, lowest tertiles; Cutoff 3, lowest quartiles; Cutoff 4, optimal point

Bold characters indicate statistically significant result

^a^Adjusted for age at diagnosis, histological grade, clinical stage, and HER2 status

The independent prognostic effects of AGR2 and FOXA1 in all 915 patients and in ER-negative ones were also explored. The cutoff points for all patients, ER-positive or ER-negative ones were defined by the corresponding sample, since the expression level of AGR2 and FOXA1 was significantly associated with ER status. Among all 915 patients, the high expression level of AGR2 was associated with a poor PFS [HR (95% CI) = 1.62 (1.09, 2.40)] with the cutoff 4 (Supplemental Table 2, see Additional file 2). Among patients with ER-negative breast cancer, the high expression level of FOXA1 was associated with a poor PFS [HR (95% CI) = 2.60 (1.15, 5.88)] with the cutoff 4 (Supplemental Table 3, see Additional file 3).

**Table 3:** Effect of AGR2 on the association between FOXA1 and ER-positive breast cancer PFS (*N* = 650)

AGR2	FOXA1	Events /Total	Crude HR (95%CI)	Adjusted HR (95%CI)^a^
Cutoff 1				
Median _low_	Median _low_	18 /133	1.00 (reference)	1.00 (reference)
	Median _high_	25 /105	**1.84 (1.01, 3.38)**	1.86 (0.99, 3.53)
Median _high_	Median _low_	34 /147	1.00 (reference)	1.00 (reference)
	Median _high_	55 /265	0.85 (0.55, 1.30)	0.84 (0.53, 1.33)
Interaction^b^			*P* = **0.039**	*P* = **0.036**
Cutoff 2				
Tertile1	Tertile1	13 /118	1.00 (reference)	1.00 (reference)
	Tertile2-3	23 /103	**2.16 (1.10, 4.27)**	**2.37 (1.14, 4.91)**
Tertile2-3	Tertile1	38 /143	1.00 (reference)	1.00 (reference)
	Tertile2-3	58 /286	0.73 (0.48, 1.09)	0.70 (0.45, 1.08)
Interaction^b^			*P* = **0.005**	*P* = **0.004**
Cutoff 3				
Quartile1	Quartile1	3 /69	1.00 (reference)	1.00 (reference)
	Quartile2-4	21 /96	**5.62 (1.68, 18.84)**	**7.24 (1.91, 27.44)**
Quartile2-4	Quartile1	29 /108	1.00 (reference)	1.00 (reference)
	Quartile2-4	79 /377	0.73 (0.48, 1.12)	0.77 (0.49, 1.20)
Interaction^b^			*P* < **0.001**	*P* < **0.001**
Cutoff 4				
Low	Low	0 /25	1.00 (reference)	1.00 (reference)
	High	14 /100	**/**	**/**
High	Low	10 /47	1.00 (reference)	1.00 (reference)
	High	108 /478	1.15 (0.60, 2.20)	1.25 (0.63, 2.49)
Interaction^b^			*P* = **0.015**	*P* = **0.013**

Cutoff 1, median; Cutoff 2, lowest tertiles; Cutoff 3, lowest quartiles; Cutoff 4, optimal point

Bold characters indicate statistically significant result

^a^Adjusted for age at diagnosis, histological grade, clinical stage, and HER2 status

^b^Models including both AGR2 and FOXA1 with and without added interaction term of AGR2 and FOXA1 (nested models) were compared using the Chi-square test

### Statistical interaction between AGR2 and FOXA1 on the PFS

In both univariate and multivariate analyses, statistical interaction between AGR2 and FOXA1 on the PFS was significant (all *P* < 0.05) based on every cutoff point for ER-positive breast cancer (Table 3; Supplemental Table 4, see Additional file 4). When compared with the expression level of FOXA1 at tertile1, the expression level of FOXA1 at tertile2-3 was associated with a poor PFS [HR (95% CI) = 2.37 (1.14, 4.91)] only in patients with the expression level of AGR2 at tertile1 but not tertile2-3 in multivariate analysis (Table 3). The expression level of FOXA1 at quartile2-4 was more significantly associated with a poor PFS [HR (95% CI) = 7.24 (1.91, 27.44)] only in patients with the expression level of AGR2 at quartile1 in multivariate analysis.

**Table 4:** Association between the combination of AGR2 and FOXA1 and ER-positive breast cancer PFS (*N* = 650)

AGR2	FOXA1	Events /Total	Crude HR (95%CI)	Adjusted HR (95%CI) ^a^
Cutoff 1				
Median _low_	Median _low_	18 /133	1.00 (reference)	1.00 (reference)
Median _low_	Median _high_	25 /105	**1.87 (1.02, 3.43)**	**1.89 (1.02, 3.52)**
Median _high_	Median _low_	34 /147	**1.83 (1.03, 3.24)**	1.76 (0.97, 3.19)
Median _high_	Median _high_	55 /265	1.57 (0.92, 2.67)	1.46 (0.83, 2.56)
Cutoff 2				
Tertile1	Tertile1	13 /118	1.00 (reference)	1.00 (reference)
Tertile1	Tertile2-3	23 /103	**2.22 (1.13, 4.39)**	**2.32 (1.15, 4.69)**
Tertile2-3	Tertile1	38 /143	**2.69 (1.43, 5.05)**	**2.65 (1.37, 5.13)**
Tertile2-3	Tertile2-3	58 /286	**1.97 (1.08, 3.60)**	1.84 (0.97, 3.48)
Cutoff 3				
Quartile1	Quartile1	3 /69	1.00 (reference)	1.00 (reference)
Quartile1	Quartile2-4	21 /96	**5.80 (1.73, 19.44)**	**6.13 (1.82, 20.71)**
Quartile2-4	Quartile1	29 /108	**7.53 (2.29, 24.71)**	**6.91 (2.08, 22.92)**
Quartile2-4	Quartile2-4	79 /377	**5.53 (1.75, 17.52)**	**5.18 (1.62, 16.55)**

Cutoff 1, median; Cutoff 2, lowest tertiles; Cutoff 3, lowest quartiles; Cutoff 4, optimal point

Bold characters indicate statistically significant result

^a^Adjusted for age at diagnosis, histological grade, clinical stage, and HER2 status

The effect of FOXA1 on the prognostic role of AGR2 was similar: the poor PFS associated with the expression level of AGR2 at tertile2-3 [HR (95% CI) = 2.61 (1.34, 5.08)] or quartile2-4 [HR (95% CI) = 6.97 (2.09, 23.24)] was observed only in patients with the expression level of FOXA1 at tertile1 or quartile1 (Supplemental Table 4, see Additional file 4). In multivariate analysis of all 915 patients or ER-negative ones, no significant statistical interaction between AGR2 and FOXA1 was found with all the cutoff points (Supplemental Table 5, see Additional file 5).

### Effect of AGR2 and FOXA1 combination on ER-positive breast cancer PFS

Effect of the combination of AGR2 and FOXA1 on the PFS was explored only in ER-positive breast cancer, since the significant statistical interaction was observed only in ER-positive breast cancer. In multivariate analysis, when defined the cutoff point by median, patients with AGR2 _low_ /FOXA1 _high_ tumors [HR (95% CI) = 1.89 (1.02, 3.52)] had a poor PFS when compared with patients with AGR2 _low_ /FOXA1 _low_ tumors (Table 4). Furthermore, patients with AGR2 _tertile1_ /FOXA1 _tertile2-3_ [HR (95% CI) = 2.32 (1.15, 4.69)] or AGR2 _tertile2-3_ /FOXA1 _tertile1_ [HR (95% CI) = 2.65 (1.37, 5.13)] tumors had a worse PFS when compared with patients with AGR2 _tertile1_ /FOXA1 _tertile1_ tumors. Importantly, patients with AGR2 _qurrtile1_ /FOXA1 _quartile2-4_ [HR (95% CI) = 6.13 (1.82, 20.71)], AGR2 _quartile2-4_ /FOXA1 _quartile1_ [HR (95% CI) = 6.91 (2.08, 22.92)] or AGR2 _quartile2-4_ /FOXA1 _quartile2-4_ [HR (95% CI) = 5.18 (1.62, 16.55)] tumors had a worse PFS when compared with patients with AGR2 _quartile1_ /FOXA1 _quartile1_ tumors. No results were shown based on the cutoff 4, since there were no events in the subgroup with AGR2 _low_ /FOXA1 _low_ tumors.

## Discussion

In our study, the expression levels of AGR2 and FOXA1 in tumor tissues were significantly higher than that in adjacent tissues and there was a positive correlation between them. The high expression level of either AGR2 or FOXA1 was associated with histological grade I/II, ER/PR-positive tumors and a poor PFS in ER-positive tumors based on the optimal cutoff point. The significant statistical interaction between AGR2 and FOXA1 on the PFS was found only in ER-positive breast cancer based on every cutoff point. Moreover, we found that patients with the combined low expression levels of AGR2 and FOXA1 had the best PFS than the rest in ER-positive breast cancer.

Consistent with our study, many previous studies have also found that a higher expression of either AGR2 or FOXA1 was related to ER/PR-positive tumors [8, 17, 19, 24, 28], which was supported by the findings that FOXA1 was required for the expression of ER-regulated genes [4, 29, 30], and AGR2 was the target of ER [31]. The positive association of AGR2 expression with HER2 status has also been reported in a previous study [18], which may be explained to some extent by that activation of HER2 leads to the activation of extracellular signal-regulated kinases 1,2 (Erk1,2) and Akt, and these kinases were involved in the up-regulation of AGR2 expression [32, 33]. Underlying mechanism of the association between these two markers and the histological grade needs to be further explored.

For the independent prognostic roles of either AGR2 or FOXA1, when defining the subgroup by the optimal cutoff point, we found that the high expression of either AGR2 or FOXA1 was associated with a poor PFS in ER-positive tumors, while there was no significant association based on the other cutoff points, which may to some extent explain the different results of previous studies in which various cutoff points were applied [3, 10, 24, 34]. In addition, our findings also showed that the smaller the cutoff value, the more obvious the association of either AGR2 or FOXA1 with the prognosis in ER-positive tumors (Table 4), suggesting that it would be helpful for improving the prognosis to reduce the expression level of either AGR2 or FOXA1.

Previous studies showed that the expression of AGR2 in breast cancer cells required the transcription factor FOXA1 [35]. In our analyses, the correlation coefficient between them in the tumor tissues was 0.441. In addition, we found that 52.5% of patients with a low expression level of FOXA1 had a high expression level of AGR2 in the ER-positive subgroup based on the cutoff point 1. Furthermore, based on the cutoff point 2 to 4, the ratio was 54.8%, 61.0% and 65.3%, respectively. These findings suggested that the expression of AGR2 in the breast cancer tissues may not be entirely dependent on FOXA1, and the potential associations need to be further explored.

There was a significant statistical interaction between AGR2 and FOXA1 on the prognosis in ER-positive tumors. The prognostic role of either of them was observed only at the low level of the other. In other words, there was a significant prognostic difference between the low AGR2/low FOXA1 subgroup and the low AGR2/high FOXA1 or high AGR2/low FOXA1 subgroup. Furthermore, our findings showed that patients with the combined low expressions of both them had the lowest risk of disease progression, and the rest had a higher risk. It has been found that FOXA1 could regulate the expression of membrane receptor LYPD3, and AGR2 was the ligand of LYPD3 [16]. Moreover, activation of the LYPD3 signaling pathway was associated with the endocrine therapy-resistant and metastasis of breast cancer [16, 36]. Together with our results, the high expression of at least one marker in AGR2 and FOXA1 could activate the LYPD3 signaling pathway and lead to a poor prognosis. Interestingly, the expression of AGR2 in breast cancer cells required the transcription factor FOXA1 [35], and our findings suggested that only decreasing the expression of FOXA1 could not entirely prevent the corresponding signal pathway. Patients with the low expression of FOXA1 and the high expression of AGR2 also needed the treatment to reduce the level of AGR2. Therefore, the combined drug therapy to reduce the expression levels of both AGR2 and FOXA1 is essential.

There were three main limitations to the present study. First, the IHC staining was evaluated by only one pathologist, which may lead to misclassification bias. However, the pathologist’s evaluation criteria are consistent; the relative relationships between different markers are almost unaffected. Second, only patients with tumor > 1 cm were included, which may lead to selective bias. However, the prognosis of patients with tumor ≤ 1 cm was excellent, even with less treatment [37]. Therefore, it is acceptable to select the patients with tumor > 1 cm. Third, we did not collect the information of treatment. However, the treatment was determined according to the clinicopathological characteristics and most of the patients would comply with the clinical treatment guideline [38]. Therefore, the adjustment of clinicopathological characteristics was able to largely control the confounding effects of the treatment.

## Conclusions

In conclusion, this study firstly demonstrated that there was a statistical interaction between AGR2 and FOXA1 on the prognosis of ER-positive breast cancer, and the combined low expression levels of both them were associated with the best prognosis than the rest. It was suggested that decreasing the expression levels of both AGR2 and FOXA1 simultaneously would be necessary to improve the prognosis of ER-positive breast cancer patients.

### Supplementary Information

**Additional file 1: Supplementary Table 1. **Univariate association between the clinicopathological characteristics and breast cancer PFS.

**Additional file 2: Supplementary Table 2. **Hazard ratios for the associations between AGR2 and FOXA1 and the PFS of all the patients (*N *=915).

**Additional file 3: Supplementary Table 3. **Hazard ratios for the associations between AGR2 and FOXA1 and ER-negative breast cancer PFS (*N *=228).

**Additional file 4: Supplementary Table 4. **Effect of FOXA1 on the association between AGR2 and ER-positive breast cancer PFS (*N *=650).

**Additional file 5: Supplementary Table 5. **Statistical interaction of AGR2 and FOXA1 on the PFS of all the patients or ER-negative breast cancer patients.

## Data Availability

The datasets used and/or analysed during the current study are available from the corresponding author on reasonable request.

## Abbreviations

AGR2: Anterior gradient 2
CI: Confidence interval
DAB: Diaminobenzidine
ER: Estrogen receptor
FOXA1: Forkhead box A1
HER2: Human epidermal growth factor receptor 2
HR: Hazard ratio
IHC: Immunohistochemistry
PFS: Progression-free survival
PR: Progesterone receptor
TMA: Tissue microarray
