# Supplementary material for: AGR2 and FOXA1 as prognostic markers in ER-positive breast cancer
Source: BMC Cancer. 2023 Aug 11;23:743. doi: 10.1186/s12885-023-10964-6 (PMC10416444; doi:10.1186/s12885-023-10964-6)
Supplement: Supplementary file 1 — Additional file 1: Supplementary Table 1. Univariate association between the clinicopathological characteristics and breast cancer PFS. [file 12885_2023_10964_MOESM1_ESM.pdf]

Supplementary Table 1 Univariate association between the clinicopathological characteristics and breast cancer PFS

Characteristics	*N* (%)	Event (%)	HR (95%CI)
Age (years)			
≤ 40	200 (21.9)	44 (22.7)	1.12 (0.79,1.58)
41-60	609 (66.6)	123 (63.4)	1.00 (reference)
> 60	106 (11.6)	27 (13.9)	1.21 (0.80,1.84)
Histological grade			
I/II	620 (73.8)	129 (72.5)	1.00 (reference)
III	220 (26.2)	49 (27.5)	1.13 (0.81,1.57)
Tumor size (cm)			
≤ 2	282 (30.8)	42 (21.6)	1.00 (reference)
> 2	633 (69.2)	152 (78.4)	**1.62 (1.15,2.28)**
Nodal status			
Negative	421 (46.0)	59 (30.4)	1.00 (reference)
Positive	494 (54.0)	135 (69.6)	**2.19 (1.63,2.98)**
Clinical stage			
I	169 (18.5)	18 (9.3)	1.00 (reference)
II	493 (53.9)	95 (49.0)	**1.81 (1.09,2.99)**
III	253 (27.7)	81 (41.8)	**3.51 (2.11,5.85)**
ER			
Negative	228 (26.0)	53 (28.6)	1.00 (reference)
Positive	650 (74.0)	132 (71.4)	0.80 (0.58,1.10)
PR			
Negative	237 (27.0)	48 (25.8)	1.00 (reference)
Positive	641 (73.0)	138 (74.2)	0.95 (0.68,1.32)
HER2			
Negative	614 (66.5)	139 (71.6)	1.00 (reference)
Equivocal	77 (8.2)	18 (9.3)	1.00 (0.61,1.64)
Positive	224 (25.3)	37 (19.1)	0.75 (0.52,1.07)

Bold characters indicate statistically significant result.
