# Supplementary material for: AGR2 and FOXA1 as prognostic markers in ER-positive breast cancer
Source: BMC Cancer. 2023 Aug 11;23:743. doi: 10.1186/s12885-023-10964-6 (PMC10416444; doi:10.1186/s12885-023-10964-6)
Supplement: Supplementary file 2 — Additional file 2: Supplementary Table 2. Hazard ratios for the associations between AGR2 and FOXA1 and the PFS of all the patients (N =915). [file 12885_2023_10964_MOESM2_ESM.pdf]

Supplementary Table 2 Hazard ratios for the associations between AGR2 and FOXA1 and the PFS of all the patients (*N* =915)

Markers	H-score	Events /Total	Crude HR (95%CI)	Adjusted HR (95%CI)[a]
AGR2				
Cutoff 1				
Median $_{low}$	0-270.0	83 /400	1.00 (reference)	1.00 (reference)
Median $_{high}$	270.0-300.0	111 /515	1.01 (0.76, 1.34)	1.09 (0.79, 1.50)
Cutoff 2				
Tertile1	0-247.5	61 /302	1.00 (reference)	1.00 (reference)
Tertile2-3	247.5-300.0	133 /613	1.05 (0.78, 1.42)	1.22 (0.86, 1.73)
Cutoff 3				
Quartile1	0-195.0	42 /225	1.00 (reference)	1.00 (reference)
Quartile2-4	195.0-300.0	152 /690	1.18 (0.84, 1.66)	1.50 (1.00, 2.25)
Cutoff 4				
Low	0-215.0	44 /247	1.00 (reference)	1.00 (reference)
High	215.0-300.0	150 /668	1.29 (0.92, 1.80)	**1.62 (1.09, 2.40)**
FOXA1				
Cutoff 1				
Median $_{low}$	0-280.0	93 /434	1.00 (reference)	1.00 (reference)
Median $_{high}$	280.0-300.0	101 /481	0.96 (0.73, 1.28)	1.08 (0.78, 1.49)
Cutoff 2				
Tertile1	0-270.0	47 /228	1.00 (reference)	1.00 (reference)
Tertile2-3	270.0-300.0	147 /687	1.02 (0.74, 1.42)	1.26 (0.85, 1.87)
Cutoff 3				
Quartile1	0-270.0	47 /228	1.00 (reference)	1.00 (reference)
Quartile2-4	270.0-300.0	147 /687	1.02 (0.74, 1.42)	1.26 (0.85, 1.87)
Cutoff 4				
Low	0-295.0	160 /780	1.00 (reference)	1.00 (reference)
High	295.0-300.0	34 /135	1.22 (0.84, 1.76)	1.21 (0.80, 1.82)

Note: Cutoff 1, median; Cutoff 2, lowest tertiles; Cutoff 3, lowest quartiles; Cutoff 4, optimal point.

[a] Adjusted for age at diagnosis, histological grade, clinical stage, ER and HER2 status.

Bold characters indicate statistically significant result.
