# Supplementary material for: AGR2 and FOXA1 as prognostic markers in ER-positive breast cancer
Source: BMC Cancer. 2023 Aug 11;23:743. doi: 10.1186/s12885-023-10964-6 (PMC10416444; doi:10.1186/s12885-023-10964-6)
Supplement: Supplementary file 3 — Additional file 3: Supplementary Table 3. Hazard ratios for the associations between AGR2 and FOXA1 and ER-negative breast cancer PFS (N =228). [file 12885_2023_10964_MOESM3_ESM.pdf]

Supplementary Table 3 Hazard ratios for the associations between AGR2 and FOXA1 and ER-negative breast cancer PFS (*N* =228)

Markers	H-score	Events /Total	Crude HR (95%CI)	Adjusted HR (95%CI)[a]
AGR2				
Cutoff 1				
Median $_{low}$	0-212.5	27 /114	1.00 (reference)	1.00 (reference)
Median $_{high}$	212.5-300.0	26 /114	0.93 (0.54, 1.59)	1.24 (0.61, 2.52)
Cutoff 2				
Tertile1	0-22.5	19 /76	1.00 (reference)	1.00 (reference)
Tertile2-3	22.5-300.0	34 /152	0.83 (0.47, 1.46)	1.04 (0.46, 2.34)
Cutoff 3				
Quartile1	0-2.5	15 /62	1.00 (reference)	1.00 (reference)
Quartile2-4	2.5-300.0	38 /166	0.88 (0.48, 1.60)	1.04 (0.47, 2.30)
Cutoff 4				
Low	0-20.0	19 /73	1.00 (reference)	1.00 (reference)
High	20.0-300.0	34 /155	0.77 (0.44, 1.36)	0.97 (0.42, 2.22)
FOXA1				
Cutoff 1				
Median $_{low}$	0-270.0	26 /111	1.00 (reference)	1.00 (reference)
Median $_{high}$	270.0-300.0	27 /117	0.93 (0.54, 1.60)	1.18 (0.55, 2.53)
Cutoff 2				
Tertile1	0-135.0	17 /76	1.00 (reference)	1.00 (reference)
Tertile2-3	135.0-300.0	36 /152	1.05 (0.59, 1.88)	1.22 (0.55, 2.72)
Cutoff 3				
Quartile1	0-10.0	14 /61	1.00 (reference)	1.00 (reference)
Quartile2-4	10.0-300.0	39 /167	0.96 (0.52, 1.77)	1.23 (0.81, 1.86)
Cutoff 4				
Low	0-245.0	19 /95	1.00 (reference)	1.00 (reference)
High	245.0-300.0	34 /133	1.29 (0.73, 2.26)	**2.60 (1.15, 5.88)**

Note: Cutoff 1, median; Cutoff 2, lowest tertiles; Cutoff 3, lowest quartiles; Cutoff 4, optimal point.

[a] Adjusted for age at diagnosis, histological grade, clinical stage, and HER2 status.

Bold characters indicate statistically significant result.
