# Supplementary material for: AGR2 and FOXA1 as prognostic markers in ER-positive breast cancer
Source: BMC Cancer. 2023 Aug 11;23:743. doi: 10.1186/s12885-023-10964-6 (PMC10416444; doi:10.1186/s12885-023-10964-6)
Supplement: Supplementary file 4 — Additional file 4: Supplementary Table 4. Effect of FOXA1 on the association between AGR2 and ER-positive breast cancer PFS (N =650). [file 12885_2023_10964_MOESM4_ESM.pdf]

Supplementary Table 4 Effect of FOXA1 on the association between AGR2 and ER-positive breast cancer PFS (*N* =650)

FOXA1	AGR2	Events /Total	Crude HR (95%CI)	Adjusted HR (95%CI) [a]
Cutoff 1				
Median $_{low}$	Median $_{low}$	18 /133	1.00 (reference)	1.00 (reference)
	Median $_{high}$	34 /147	**1.80 (1.02, 3.19)**	1.81 (0.99, 3.31)
Median $_{high}$	Median $_{low}$	25 /105	1.00 (reference)	1.00 (reference)
	Median $_{high}$	55 /265	0.82 (0.51, 1.32)	0.75 (0.46, 1.23)
Interaction [b]			*P* =**0.039**	*P* =**0.036**
Cutoff 2				
Tertile1	Tertile1	13 /118	1.00 (reference)	1.00 (reference)
	Tertile2-3	38 /143	**2.65 (1.41, 4.98)**	**2.61 (1.34, 5.08)**
Tertile2-3	Tertile1	23 /103	1.00 (reference)	1.00 (reference)
	Tertile2-3	58 /286	0.88 (0.54, 1.43)	0.78 (0.48, 1.29)
Interaction [b]			*P* =**0.005**	*P* =**0.004**
Cutoff 3				
Quartile1	Quartile1	3 /69	1.00 (reference)	1.00 (reference)
	Quartile2-4	29 /108	**7.19 (2.19, 23.63)**	**6.97 (2.09, 23.24)**
Quartile2-4	Quartile1	21 /96	1.00 (reference)	1.00 (reference)
	Quartile2-4	79 /377	0.95 (0.59, 1.54)	0.85 (0.52, 1.40)
Interaction [b]			*P* <**0.001**	*P* <**0.001**
Cutoff 4				
Low	Low	0 /25	1.00 (reference)	1.00 (reference)
	High	10 /47	/	/
High	Low	14 /100	1.00 (reference)	1.00 (reference)
	High	108 /478	**1.75 (1.00, 3.05)**	1.67 (0.95, 2.94)
Interaction [b]			*P* =**0.015**	*P* =**0.013**

Note: Cutoff 1, median; Cutoff 2, lowest tertiles; Cutoff 3, lowest quartiles; Cutoff 4, optimal point.

[a] Adjusted for age at diagnosis, histological grade, clinical stage, and HER2 status.

[b] Models including both AGR2 and FOXA1 with and without added interaction term of AGR2 and FOXA1 (nested models) were compared using the Chi-square test.

Bold characters indicate statistically significant result.
