# Supplementary material for: AGR2 and FOXA1 as prognostic markers in ER-positive breast cancer
Source: BMC Cancer. 2023 Aug 11;23:743. doi: 10.1186/s12885-023-10964-6 (PMC10416444; doi:10.1186/s12885-023-10964-6)
Supplement: Supplementary file 5 — Additional file 5: Supplementary Table 5. Statistical interaction of AGR2 and FOXA1 on the PFS of all the patients or ER-negative breast cancer patients. [file 12885_2023_10964_MOESM5_ESM.pdf]

Supplementary Table 5 Statistical interaction of AGR2 and FOXA1 on the PFS of all the patients or ER-negative breast cancer patients

AGR2	FOXA1	Adjusted HR (95%CI)[a]	
		All	ER-negative
Cutoff 1			
Median $_{low}$	Median $_{low}$	1.00 (reference)	1.00 (reference)
	Median $_{high}$	1.17 (0.73, 1.89)	1.72 (0.54, 5.49)
Median $_{high}$	Median $_{low}$	1.00 (reference)	1.00 (reference)
	Median $_{high}$	0.84 (0.56, 1.26)	0.89 (0.31, 2.57)
Interaction [b]		0.375	0.526
Cutoff 2			
Tertile1	Tertile1	1.00 (reference)	1.00 (reference)
	Tertile2-3	1.14 (0.63, 2.06)	1.64 (0.53, 5.07)
Tertile2-3	Tertile1	1.00 (reference)	1.00 (reference)
	Tertile2-3	1.00 (0.61, 1.62)	2.69 (0.56, 12.85)
Interaction [b]		0.771	0.570
Cutoff 3			
Quartile1	Quartile1	1.00 (reference)	1.00 (reference)
	Quartile2-4	1.23 (0.60, 2.54)	0.78 (0.21, 2.96)
Quartile2-4	Quartile1	1.00 (reference)	1.00 (reference)
	Quartile2-4	0.95 (0.61, 1.49)	2.52 (0.52, 12.35)
Interaction [b]		0.786	0.271
Cutoff 4			
Low	Low	1.00 (reference)	1.00 (reference)
	High	0.97 (0.28, 3.32)	3.36 (0.96, 11.73)
High	Low	1.00 (reference)	1.00 (reference)
	High	1.09 (0.72, 1.67)	2.30 (0.71, 7.43)
Interaction [b]		0.798	0.442

Note: Cutoff 1, median; Cutoff 2, lowest tertiles; Cutoff 3, lowest quartiles; Cutoff 4, optimal point.

[a] Adjusted for age at diagnosis, histological grade, clinical stage, and HER2 status.

[b] Models including both AGR2 and FOXA1 with and without added interaction term of AGR2 and FOXA1 (nested models) were compared using the Chi-square test.

Bold characters indicate statistically significant result.
